# Rapid, Tunable, and Scalable Patterning of Plasmonic Films for Biosensing Applications

**DOI:** 10.1021/acsami.5c09086

**Published:** 2025-09-01

**Authors:** John H. Molinski, Junhu Zhou, Tim Palinski, John X.J. Zhang

**Affiliations:** † Thayer School of Engineering, 145792 Dartmouth College, 15 Thayer Drive, Hanover, New Hampshire 03755, United States; ‡ Honeywell International Inc, 303 S Technology Ct, Broomfield, Colorado 80021, United States; § Norris Cotton Cancer Center, Dartmouth Hitchcock Medical Center, 1 Medical Center Dr, Lebanon, New Hampshire 03766, United States

**Keywords:** Laser-induced plasmonic structures, Large-scale patterning, Plasmon-enhanced fluorescence, Surface-enhanced Raman scattering, Plasmonic biosensing

## Abstract

Large-scale and rapid fabrication of tailorable, uniform, and patterned plasmonic surfaces is of significant interest within the biosensing application space. Prior attempts have primarily investigated solution and thin film-based methods, which often incorporate complex synthesis procedures or high-temperature processes, limiting scalability and tunability of the optical response. Herein, we present a simple two-step fabrication method that enables the fabrication of large-scale (>in^2^), rapid (<10 min/in^2^), and uniformly sized (polydispersity index < 0.2) plasmonic nanoparticles using a rapid thin film dewetting followed by laser restructuring process. Using the fabricated films, we demonstrate tunability in particle size and packing density by altering laser processing conditions and characterize broadband electromagnetic enhancement using finite difference time domain (FDTD) simulations. With the rationally designed films, we demonstrate applicability for biosensing using plasmon-enhanced florescence within a well plate format and surface-enhanced Raman scattering, therefore showing the utility and versatility of our fabricated films.

## Graphical abstract

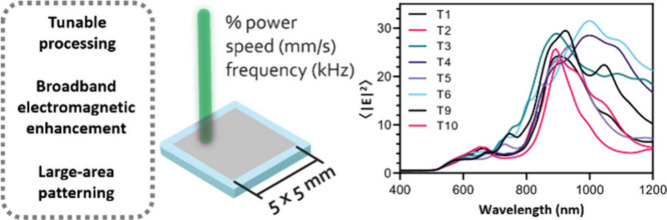

## 1 Introduction

Plasmonic nanostructures have found many applications within biosensing, with the goal to enhance sensitivity and lower the limit of detection within biological assays.^1–4^ Due to their ubiquity within research and clinical workflows alike, such advancements could immediately offer widespread applicability, offering a plug-and-play enhancement to existing assays. Recently, plasmonic nanostructures have become a particularly appealing tool within biosensing applications, with effort dedicated to optimizing nanostructure geometry and/or arrangement to maximize enhancement factors.^5^ These complex configurations, however, have come at the expense of fabrication complexity, and often nanofabrication methods are employed that necessitate specialized systems, significant fabrication times, and struggle to scale to meet the demands of biological assays.

Fundamentally, the plasmonic effect arises from the highly localized electromagnetic field enhancement stemming from the resonance coupling of incident photons with surface plasmons within a metal. Research developments have successfully utilized this phenomenon within several biosensing configurations, including at length within plasmon-enhanced fluorescence (PEF) and surface-enhanced Raman scattering (SERS), as well as within laser desorption/ionization mass spectrometry (LDI-MS) and surface plasmon resonance microscopy (SPRM).^6–8^ For PEF, SERS, and LDI-MS applications, this results in an enhanced emission or signal amplification that lowers the limit of detection, providing more sensitive assays.^9^ Surface-enhanced resonance microscopy, however, relies on interferometric imaging arising from the interference between reflective light and scattered surface plasmons.^6,10^ In all cases, however, this nanoscale phenomenon is a near-field effect, and for PEF and SERS, it occurs when the spacing between the metal and target biomolecule is on the order of 10 nm.^9,11,12^

For PEF, the magnitude of this enhancement depends on numerous (often tunable) parameters including structure/particle size,^14,15^ arrangement/geometry of structures/particles,^4,11,16^ structure/particle density,^14^ morphology, material,^19^ and spacing between fluorophore and metal. Similar with SERS applications, there are many different plasmonic enhancement strategies incorporating novel structure/particle unit cells or arrangements of the like, with substantial electric field enhancement demonstrated experimentally.^16,20^ Such plasmonic substrates have been fabricated using methodologies including nanofabrication,^2,15,21^ thermal dewetting,^23,24^ dispersion of chemically synthesized single nanoparticles,^4,11,14^ and solution-based seeding/growth.^1^ Despite these advances, the ability to finely control these parameters to maximize enhancement greatly depends on the fabrication methods utilized, where a trade-off between scalability and tunability often arises. Traditional nanofabrication methods (e.g., electron beam lithography or focused ion beam milling) offer unmatched levels of control and structure uniformity at the expense of scalability. In contrast, for particle-based methods, scalability is often high at the expense of controlled arrangement and uniformity.

Concerning fluorometric assays, robust optimization of dispersed single particles has been a promising recent development due to the ease of scalability and integration with traditional assays.^11^ The maximum field enhancement within these systems correlates highly with the localized surface plasmon resonance (LSPR)^11^ of the particle unit cell due to the dispersed particles behaving as individual noninteracting particles.^26^ This enables high levels of tunability across the fluorescence spectrum by simply changing the base particle size or shape. In contrast, for fractal-like geometries and dense particle films, the highest levels of field enhancement occur at wavelengths beyond the LSPR of the structure,^1,26,27,28^ arising from confined and localized optical excitations or “hotspots” generated by the closely packed yet distinct clusters or particles of near-percolation films. Such films have found significant applications within SERS and PEF applications alike, offering a convenient and large-area sensing surface which benefits biological sensing.^1,27^ Patterning of such films, however, necessitates microfabrication techniques such as lithography and etching, and such films have a limited range of tunability, achieved by slight changes to otherwise rigid processing conditions.

Herein, we present a novel methodology to realize large-scale (in^2^), densely packed, and tunable plasmonic nanoparticle films using a simple and scalable two-step fabrication approach and demonstrate biosensing utility within PEF and SERS applications. Following an initial metallic deposition, we demonstrate that a simple torch process can generate a consistent nanostructured substrate via dewetting of the gold thin film without the need for specialized equipment, while subsequent lasing enables tuning of the nanoparticle morphology and subsequent optical properties. Finite difference time domain (FDTD) simulations of these films provided insight into their optical properties and guided biosensing applications. With the rationally designed films, biosensing was demonstrated within PEF and SERS alike, demonstrating <1 nM sensitivity.

We believe this method will be a meaningful advancement to the field due to (1) the ability to fabricate large-area films with tunable plasmonic properties in an inexpensive and scalable manner and (2) the unique optical properties of the fabricated films, which result in broadband field enhancement, allowing for substrates to be applicable within a wide range of biological assays. We have thoroughly characterized the processing parameters and have identified key factors that influence the morphology (laser power and speed) as well as those that are robust to changes in processing (torch-induced dewetting). With optimized processing parameters, we have shown high levels of uniformity sample to sample, as well as between samples and reproducibility between batches, echoed in PEF and SERS experiments.

## 2 Results and Discussion

### 2.1 Tunable Laser-Induced Gold Nanostructure Formation

Thermal dewetting of metallic thin films using rapid thermal annealing (RTA) has been accomplished for a variety of metallic films including gold, silver, platinum, and palladium as well as alloys of each of these metals.^30–33^ The choice of metal can depend on several choices, including desired properties, cost, ease of coating, and processing requirements. Of these, gold has arisen as an intriguing material due to the resultant optical properties in the visible regime that enable facile integration with existing optics setups and characterization tools (e.g., fluorophores and dyes). Generalizing fabrication as it relates to gold thin films, there have been several processing parameters robustly associated with specific structure size and density, with distinct optical and plasmonic properties. Of these trends, conclusively it has been shown that increasing metal thin film thickness results in increases in particle size and decreases in particle packing density.^34–36^ Thus, to achieve particle formation and packing density beneficial for plasmonic properties, finely tuned ultrathin metal thin films are required, below ∼10 nm in thickness.^23^ This fact inherently limits control over several important factors mentioned that govern plasmonic enhancement, such as the ability to control packing density, particle size, and morphology, independently of one another. Similarly, the formation of gold nanostructures from the lasing of metallic thin films has been explored and mechanistically is akin to rapid thermal annealing yet is induced by high power laser pulses to generate localized dewetting of near-percolation thin films.^37,38^

Here, we achieved tunable laser-induced gold nanostructure formation using a dual-step process that first implements a simple and cost-effective rapid torch treatment for gold thin film dewetting followed by laser-induced restructuring to form optically tunable and highly uniform nanoparticle arrays (Figure 1). Initial dewetting was shown to be achievable in ambient conditions, under no vacuum, and without the need for special environments or equipment beyond a fume hood for safety (see Materials and Methods for the detailed experimental setup). Moreover, the laser used was a compact and accessible benchtop system (Technifor Laser Marking Machine LW1), without the need for specialized equipment or processing. For both torch and laser processing, the sample was placed perpendicular to the source, at working distances of 100 and 194 mm, respectively. Importantly, for all large-area sample processing (>10 mm^2^), we recommend the use of quartz glass due to its low thermal expansion, which prevents unexpected fracturing of the substrate following heat treatment.

**Figure 1:**
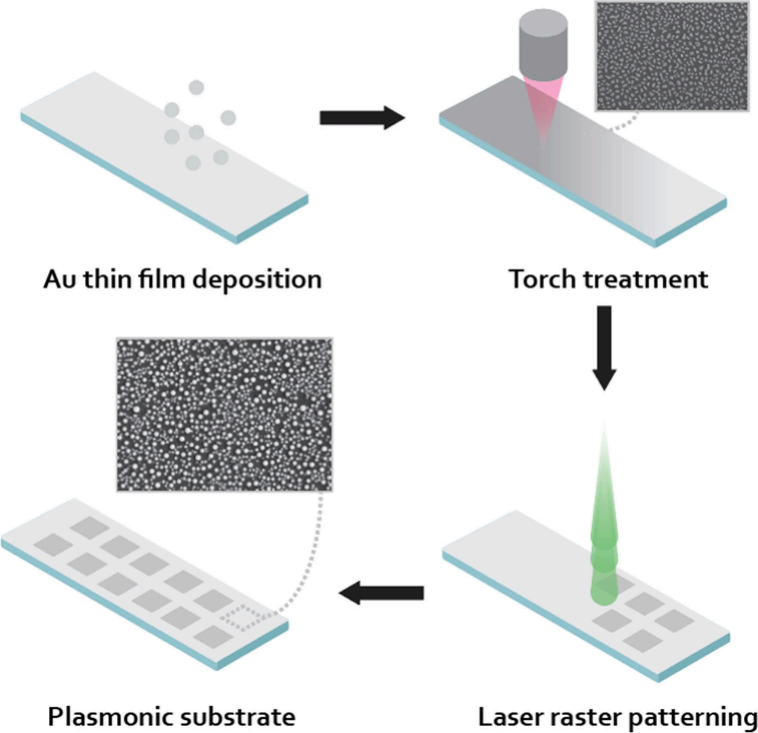
Fabrication schematic for gold nanoparticles via a laser and torch process. A thin gold layer (∼15 nm) is sputter deposited onto a glass slide, followed by torching the substrate (6–8 passes) on the back side of the glass slide. Following the torch process, the laser process is performed on the back side of the glass slide to complete particle formation.

When comparing results of this dual-step process (Figure 2A–H) to laser processed films without initial torch-induced dewetting (i.e., near-percolation sputtered thin films, shown in Figure S1), we see significant differences in average particle size and particle size distribution. Additionally, regardless of the laser processing parameters selected, laser processing of sputtered films without torch treatment was typically accompanied by particle sparsity and a lack of uniformity within patterned areas. In contrast, in films that were first subjected to a brief (<10 s) torch dewetting treatment, particle uniformity was greatly increased, and they had a higher packing density and lower mean particle size, as shown in Figure 2A–H, highlighting the ability to tune both particle size and packing density. Using this process, we have shown the ability to effectively eliminate the particle size and thin film thickness dependencies common with RTA-treated films, which limit packing density and plasmonic enhancement. Therefore, in this study, we present highly uniform and densely packed gold nanoparticle coatings from thin films significantly thicker (∼37 nm) than those of prior studies, shown to benefit plasmonic enhancement greatly.

**Figure 2:**
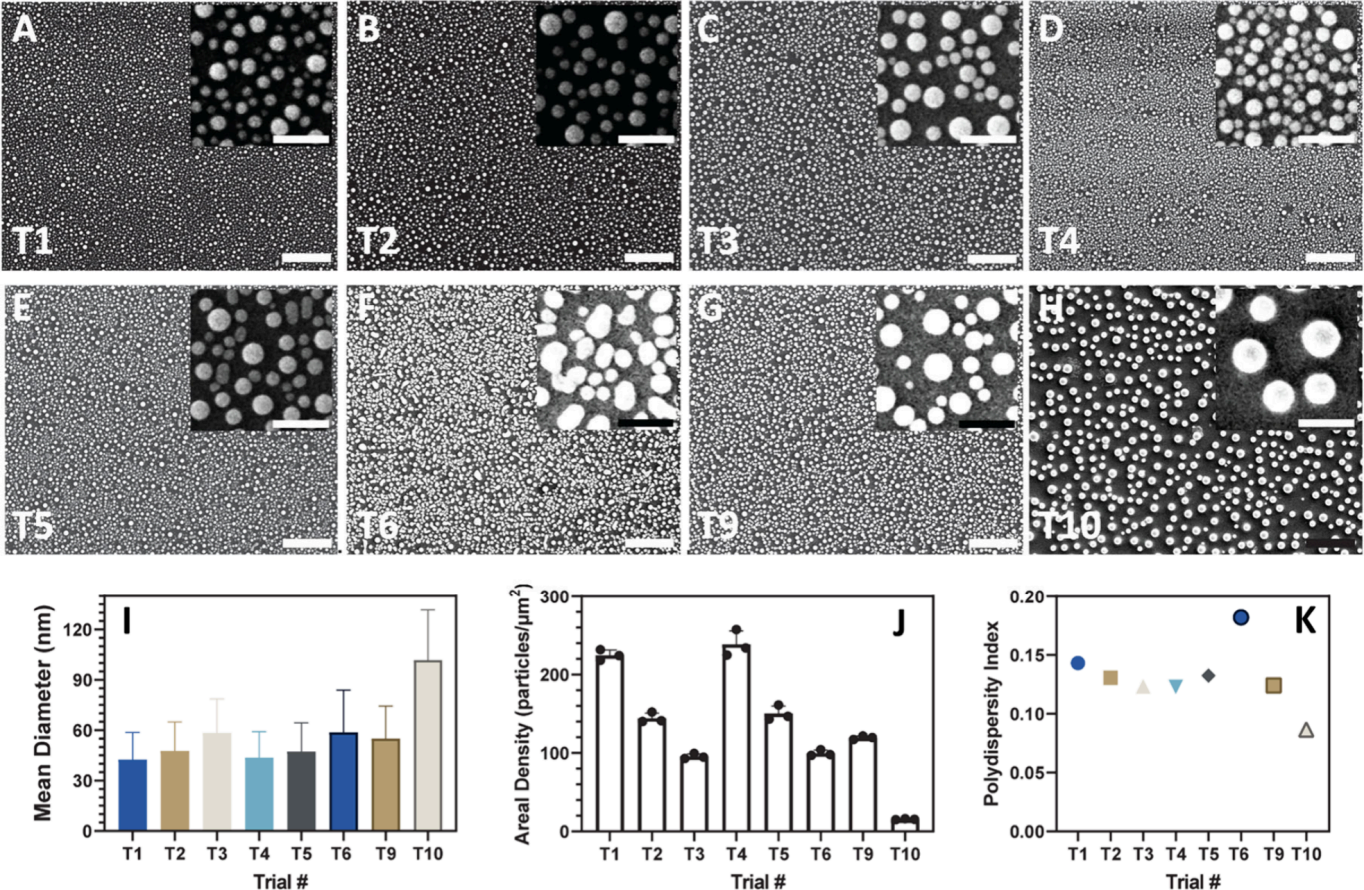
Characterization of the laser process via scanning electron microscopy and image processing analysis. (A–H) T1, T2, T3, T4, T5, T6, T9, and T10 laser processing conditions, showing scanning electron microscopy images highlighting the morphology of each case. All main image scale bars are 1 μm, whereas all inset images have a scale bar of 200 nm. (I) Mean diameter of each of the particles shown within the SEM images and (J) corresponding areal density in particles/μm^2^. (K) Polydispersity index of the various parameters analyzed, which is below the widely acknowledged 0.20 threshold for all.

As a benchmark for initial dewetting, we performed control studies using various RTA processed films to examine the differences between sputtered films and those that were torch-treated and found similar morphologies (Figure S2), an indication that torched films similarly undergo a spinodal decomposition process.^39,40^ In both films, as is well characterized for RTA-treated films, thermal treatment resulted in distinctly isolated nanostructure formation, with the previously reported trend correlating thicker films with larger particles maintained, which was also seen after laser treatment (Figure S3). With this information, a goal of laser-induced restructuring was to provide a means to control particle density while allowing for thicker films to be utilized to benefit from the plasmonic enhancement expected from thicker films^41^ resulting from increased “hotspot” formation. Stated differently, following initial dewetting, there is a substantial step change in film height because of the decomposition process, after which only restructuring occurs following laser processing. Toward this goal, different laser processing conditions were explored, including scanning speed (mm/s), laser power (%), and pulse frequency (Hz), with the resultant films characterized for their morphology and optical characteristics. Given the mechanism of fabrication, we suspect this approach to be readily applicable to the processing of alternative metallic thin films that are suitable for RTA processing, such as silver and aluminum.

### 2.2 Nanoparticle and Film Characterization

To characterize the morphology, scanning electron microscopy was completed on all nanoparticle films to investigate the effects of laser processing conditions, a full table of which is shown in Table S1. Following image collection, the overall particle size, size distribution, and polydispersity were determined using ImageJ analysis (Figure S4). Processed microscopy images are summarized in Figure 2A–H, along with the particle size distribution histograms in Figure S5. As can be seen within the images between the various trials tested, we see distinct differences in both particle size and packing density. To characterize this, we the examined mean diameter (Figure 2I) as well as defined a metric of areal density with units of particles/μm^2^ (Figure 2J), which allows for quantitative analysis and exploring of the packing density of fabricated films.

Compared with sputtered films using the same laser processing conditions, the particles formed from the dual torch and laser process are highly uniform, as indicated by the polydispersity index calculated (Figure 2K) for each of the trials tested. We suspect that introducing a torch treatment and dewetting the film prior to lasing lowers the energy required for further film decomposition, thereby resulting in more uniform and, on average, smaller particles. Considering that prior reports have shown links between increased particle packing density and higher plasmonic enhancement,^14^ we then suspected that the ability to control particle density and spacing could lead to tunable and enhanced plasmonic properties.

Following baseline optical characterization, we then sought to characterize the effect of differences in both torch treatment time and gold thickness on the resultant particle formation to determine the robustness of the dual process. In the former case, torch treatment was administered in pulses, ranging from 4 to 12 pulses in 2 pulse increments. As can be seen from the spectra in Figure S6, though there are marked differences in the base torch film, after the laser treatment using the same processing conditions (T4 selected), the spectra are nearly identical, showing robustness of the process to slight differences in initial processing conditions. Beneficially, the optical and spectral characteristics of gold nanoparticles have been characterized at length in prior studies.^42,43^ Of interest to this study is the correlation of nanoparticle size and resonance peak, given the well-known red shift of peak location present with increasing nanoparticle size.

To undertake characterization of the fabricated films, we collected absorbance spectra on torched samples, exploring spectra as a function of initial deposition time, without and with subsequent laser restructuring treatment (T4). First, the thicknesses of the resultant films were characterized using profilometry (Figure 3A), showing a linear increase of film thickness with increasing deposition time and a step change following torch treatment, indicating a restructuring process. Despite unique spectra arising following torch treatment (Figure 3B), following the same laser treatment (T4), we see little variation in the spectra irrespective of base film thickness (Figure 3C). Next, we explored differences in lasing conditions, exploring films following sputtering without the torch process (Figure 3D) and those that underwent the torch process prior to lasing (Figure 3E). Interestingly, optical characterization elucidated significant heterogeneity in the absorbance spectra for samples that omitted the initial torch process (Figure 3D), which was largely rectified in the dually processed films, which exhibited a significantly narrower resonance peak (Figure 3E). Laser processing conditions that were altered included changes to power (%), speed (mm/s), and frequency (kHz) (Figure 3F), and a full table of parameters chosen is included in the Supporting Information (Table S1). Interestingly, the resonance peak of the nanoparticles formed has a distinctive and sharp resonance at ∼540 nm, correlating well with prior literature for gold nanoparticles of equivalent sizes. However, unique to the prepared films is the close-packed nature of the particles, which may allow for hotspot generation between them, benefiting PEF and SERS applications, a facet discussed and explored in detail in the next section.

**Figure 3:**
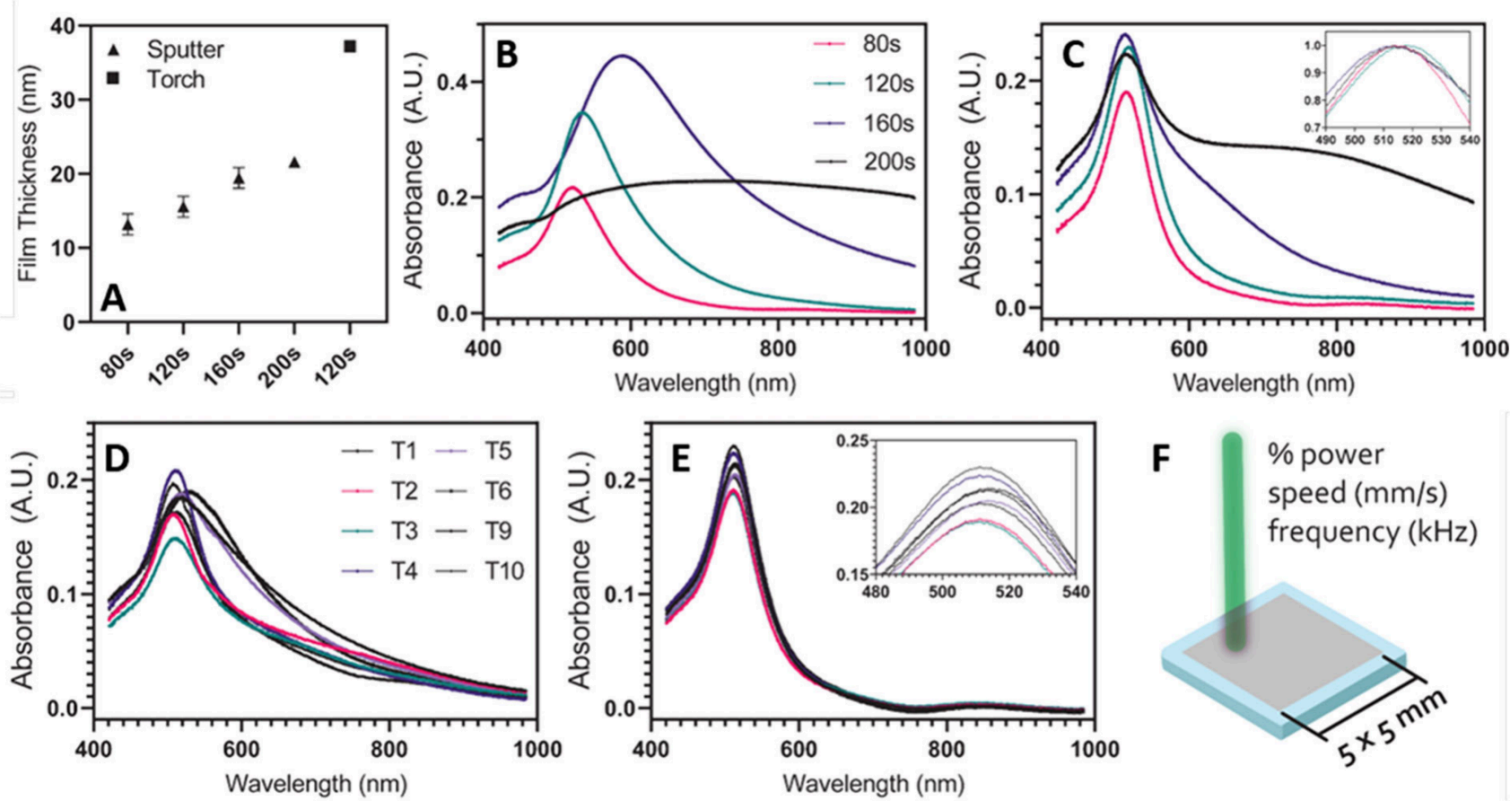
Optical characterization of torch versus no torch (sputter only) treatment. (A) Profilometry measurements of base sputtered films and of a film following torch processing following a 120 s sputter deposition. (B) Spectrometer measurements of torch-treated film for various deposition times and (C) spectrometer measurements of torched and lased (T4) samples, also for the same deposition thickness times. (D) Absorbance spectra for each of the eight laser conditions tested for sputtered and lased films and (E) those for sputtered, torched, and lased samples, both under the same initial sputtering conditions. (F) Schematic showing the three parameters modified throughout the eight laser conditions as well as the sample size used within the study.

### 2.3 Simulation Analysis and Film Optimization

Prior research on close-packed nanoparticle films like those fabricated herein has shown the potential for broad field enhancement within the visible and near-IR regimes,^1,27^ enabling intriguing applications within PEF and SERS. To expand upon the initial characterization and explore these avenues, we conducted full-wave electromagnetic simulations using the FDTD method (Lumerical, Inc.) to investigate near-field enhancement. First, we converted the SEM images from several of the trials to binary images and imported them into the Lumerical model, where the electric field profiles were calculated. The particle morphology was extruded in the *z*-direction to a thickness of 35 nm, consistent with experimental measurements (see Figure S7 for details on the model). A normally incident plane wave from the top side (air half-space) polarized in the *x*-direction (horizontal axis) was used for illumination. Periodic boundary conditions were used in the *x*- and *y*-directions, whereas perfectly matched layers were applied at *z*-boundaries. The *xy* extent of the simulation domain was 500 × 500 nm, with additional simulations completed using 250 × 250 nm windows to reduce computational time. The spatially averaged field intensity ⟨|*E*|^2^⟩ from simulations corresponds to the enhancement factor (EF), given the input field intensity *E*_0_ = 1 for all simulations (EF = |*E*|^2^/|*E*_0_|^2^). This was chosen to be large enough to contain a statistically relevant sampling of the nanoparticles while being small enough to maintain reasonable computation times.^28,44^

Figure 4A–H shows the field enhancement maps for trials T3, T4, T6, and T10 (left to right) at two different wavelengths λ = 812 nm (Figure 4A–D) and λ = 1046 nm (Figure 4E–H) for 500 × 500 nm windows. Additional simulations using 250 × 250 nm windows completed for each lasing condition are shown in Figure S8. For a given structure, the hotspot location and intensity vary significantly with the wavelength. To better quantify this effect, we calculated the spatially averaged field intensity for each surface as a function of wavelength, producing the field enhancement spectra plot in Figure 4I. As shown here, the densely packed particles in samples T3, T4, and T6 produce the highest average field enhancement, due to the multitude of small gaps/hotspot regions. In addition, these samples have broadband enhancement peaks, covering 800–1200 nm in bandwidth. This behavior is consistent for closely packed nanospheres, as reported by Shalaev,^26^ and enables a promising substrate for field-enhanced spectroscopies and fluorescence imaging over a wide range of wavelengths. In contrast, the sparse T10 sample has a relatively narrow enhancement feature, peaking near 900 nm, which offers less flexibility for practical applications. Figure 4J,K shows the average field enhancement for each of the trials at the two different wavelengths of 812 and 1046 nm. For biosensing applications using PEF and SERS, often wavelengths are in the visible regime, and thus, we additionally explored enhancement at λ = 562 nm for select parameters (T3, T4, T6, T10), which showed lower expected enhancement (∼2.4×) compared to the near-IR (Figure S9).

**Figure 4:**
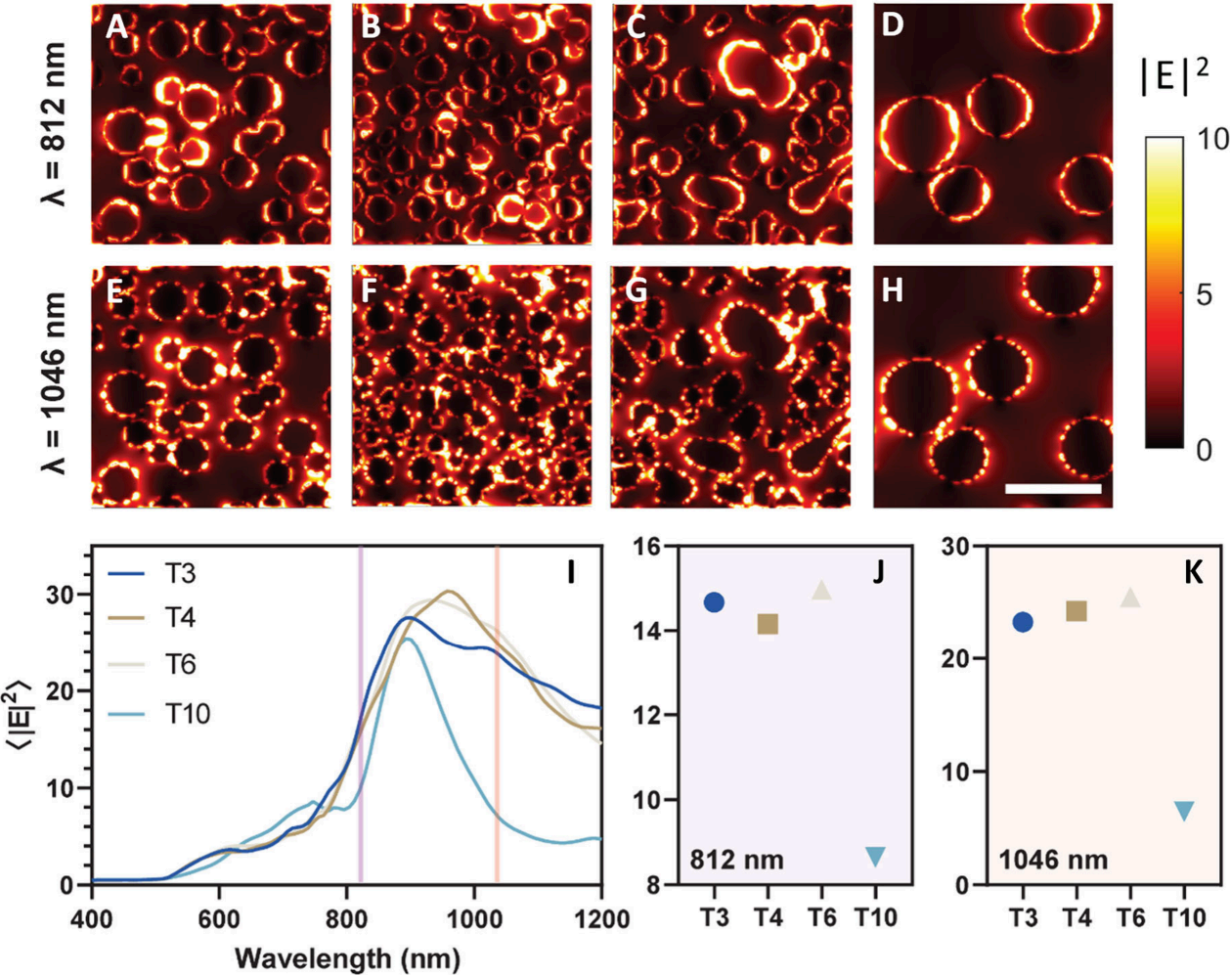
FDTD simulations of electric field enhancement via selected parameters. (A–H) Simulation images showing four selected laser processing conditions (left to right: T3, T4, T6, and T10) at two different wavelengths (top row: 812 nm, bottom row: 1046 nm). (I) Electric field enhancement versus wavelength plots for selected parameters and (J, K) the corresponding enhancement at the two specific wavelengths 812 and 1046 nm. Scale bar: 200 nm.

To further differentiate the fabricated films, lastly, we simulated a near-percolation gold thin film (5 nm) that represents close-packed “nanoislands”, rather than a close-packed particle film, which are known to exhibit strong near-IR enhancement.^1^ Figure S10 shows field enhancement over the visible and near-IR regimes, along with simulated reflectance, transmittance, and absorption spectra. In these spectra, we see the loss of the distinct, sharp resonance peak common within gold nanoparticles, along with a primary enhancement primarily within the near-IR regime. Thus, we conclude that the fabricated substrates correspond strongly, both spectrally and in resonance peak location, to closely packed uniform particles, which is confirmed from microscopy images.

### 2.4 Film-Enabled Plasmon-Enhanced Fluorescence and Surface-Enhanced Raman Scattering

Encouraged by the simulation results, we investigated the applicability of the fabricated plasmonic surfaces within biosensing applications using PEF and SERS. First, in progressing to experimental validation, we down selected to single laser processing conditions (T4) due to the high areal packing density, uniform particle size, and promising simulation results suggesting broad field enhancement. For PEF specifically, the spacing between the fluorophore and metallic surface has been demonstrated as a critical consideration governing overall enhancement.^11,14^ To finely control this spacing, prior studies have examined the use of a variety of materials including deposited thin film oxides^45^ and solution-based polymer^11,12^ depositions, showing that the highest enhancement occurs when the spacing is between 5 and 20 nm.^11,45,48^ In this study and due to the close-packed nature of the nanoparticles, we elected to explore an aluminum oxide (Al_2_O_3_) spacer deposited using atomic layer deposition, which represents a highly conformal and inert layer while also providing a suitable substrate for biosensing studies due to its robustness to biofouling.^49^ The thickness was controlled in ∼4 nm increments by increasing the number of cycles during atomic layer deposition (ALD) processing (16, 48, 80, 112, 144), which showed a highly linear and uniform increase in coating thickness from 5 to 20 nm (Figure S11), as desired. Given the profilometry results and expected results from simulations, this coating thickness was chosen to encapsulate the ∼35 nm high structures while still providing a conformal coating that allowed for the slight variations in particle size shown in microscopy images. Furthermore, this coating provided robust protection of the nanoparticle surface and provided the ability for films to be cleaned and regenerated for multiple measurements, whereas uncoated films would quickly deteriorate. To confirm that no significant changes to particle morphology arose following ALD processing, T4 processed films following Al_2_O_3_ deposition were imaged using scanning electron microscopy, which indeed showed no significant changes to particle size or distribution (Figure S12).

Two fluorophores were utilized in this study to explore PEF and biosensing applications, namely IR-125 (Exciton, Dayton, Ohio, USA, 774 nm/825 nm Ex/Em) and streptavidin labeled with Alexa Fluor 532 dye conjugate (Invitrogen, 534 nm/553 nm Ex/Em), to explore broad field enhancement within both the near-IR and visible regimes. For all tests, T4 plasmonic films were generated on fused quartz discs (Quartz Scientific, Polished disc, 15 mm diameter, 1.5 mm thickness), where they were subsequently coated with Al_2_O_3_ coatings of varying thicknesses using ALD before the fluorophore was added. A schematic of the fabrication and experimental testing is shown in Figure 5A. First, we sought to explore the enhancement as a function of spacer thickness, akin to that achieved in previous studies using polymer spacers.^11,14^ Toward this, we explored enhancement of a 1 μM IR-125 solution in dimethyl sulfoxide to elucidate enhancement as a function of Al_2_O_3_ spacer thickness (Figure 5B,C). Corresponding well with prior literature and our simulation studies, the highest enhancement occurs at a spacer thickness of ∼15 nm (Figure 5B,C), where enhancements of ∼11× were seen.

**Figure 5:**
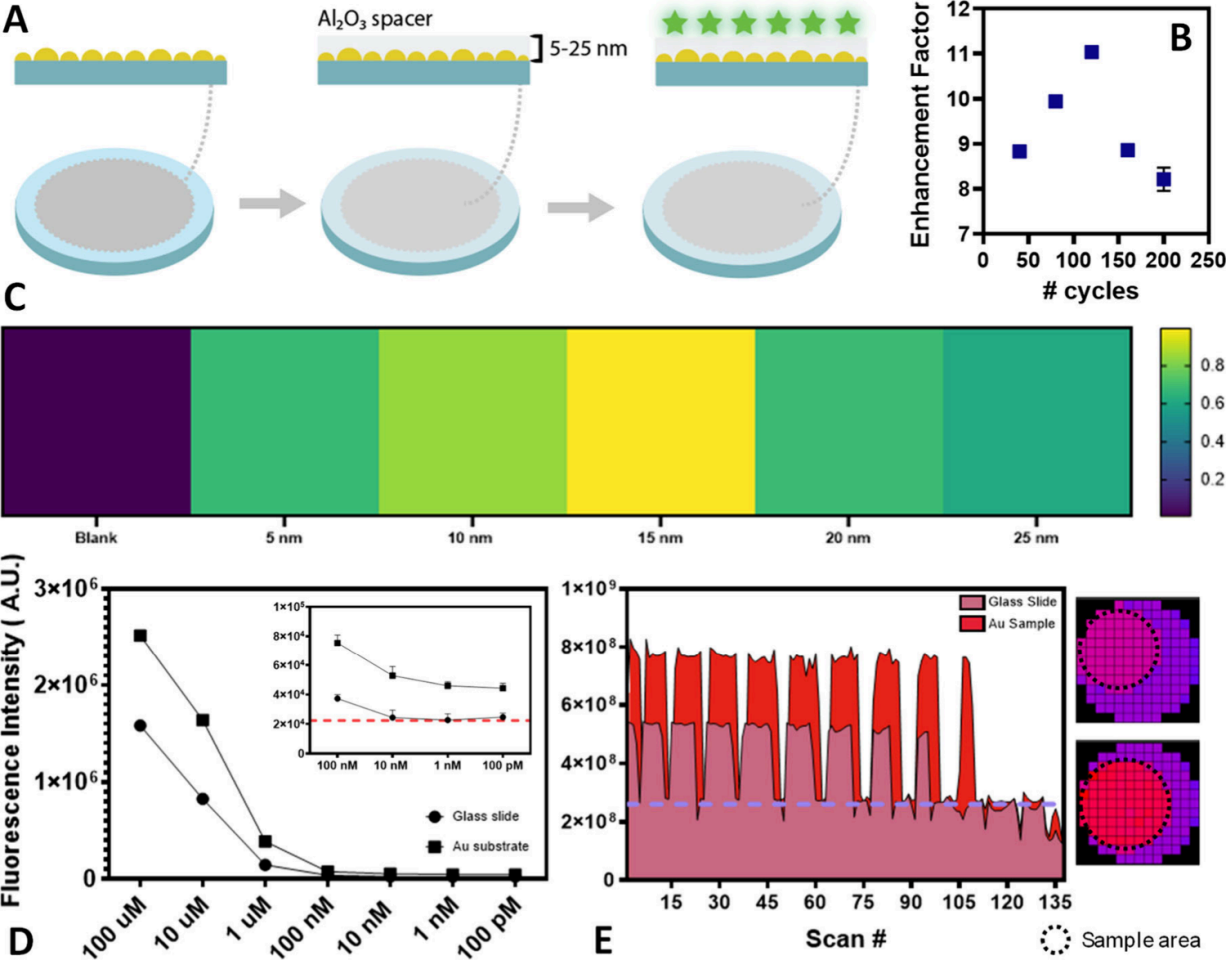
Plasmon-enhanced fluorescence using laser processed gold films. (A) Schematic of laser processed samples, using T4 laser processing parameters, with a nanoscale aluminum oxide layer on the surface. (B) Fluorescence enhancement factor as a function of atomic layer deposition cycles. (C) Heatmap representation of normalized fluorescence enhancement as a function of aluminum oxide layer thickness, with color representing the average intensity from *n* = 3 technical replicates. (D) Limit of detection of IR-125 on a T4 laser processed substrate. (E) Enhancement uniformity via well-scanning measurements of circular T4 laser processed samples for the detection of streptavidin labeled with Alexa Fluor 532 dye conjugate within a 16-well plate, where the blue line represents the background well signal, without fluorophore. All measurements were completed in *n* = 3 technical replicates with error bars calculated as the standard deviation of the measurements. For well-scanning measurements, each scan # represents an individual measurement, and results are reported without averaging.

Following preliminary validation, we progressed to explore the limit of detection using IR-125, comparing T4 laser processed samples with a glass slide control containing fluorophores in equivalent concentrations. This study explored concentrations ranging from 100 μM to 100 pM, the results of which are shown in Figure 5D, which showed notable enhancement down to a concentration of 100 pM. In contrast, the fluorescent control showed no discernible difference from a glass slide background, shown as a dotted red line within the figure inset. Lastly, for PEF exploration, we proceeded to perform a mock biosensing experiment using streptavidin labeled with Alexa Fluor 532 dye conjugate bound to the surface of the gold thin film using a biotin-PEG-thiol (PG2-BNTH-5k, Nanocs) linker. Following binding, streptavidin was incubated at a concentration of 100 ng/mL before unbound streptavidin was washed away, and it was inserted within a 24-well plate and measured within a fluorescent plate reader in well-scan mode. The results are shown in Figure 5E for both the laser processed sample and a glass slide control, showing an enhancement in the visible regime, albeit muted, which corresponds well with the simulation results. The purple line and corresponding purple squares in the heatmap represent the background signal of the well. As can be seen, there is a highly uniform enhancement over the entirety of the sample area (*d* = 15 mm in this study), which lends strong evidence for large-scale sample uniformity hinted at in the microscale polydispersity characterization.

Next, we explored the applicability of nanoparticle films for surface-enhanced Raman spectroscopy using T4 processed films of discs with optimized spacer thickness. Raman spectroscopy provides a label-free and rapid method for detecting organic molecules. In this study, a surface-enhanced Raman spectroscopy (SERS) approach was employed to quantitatively measure the Trypan blue (TB) concentration. Aqueous TB solutions were applied to the substrate with 5 μL of the analyte solution deposited onto the film. The drop, averaging ∼2 mm in diameter, was then dried in an oven at 35 °C. Using a high numerical aperture (100×) objective minimized adsorption dynamics, dilution effects, and cross-contamination while ensuring no physical contact with the sample. To mitigate bias from “coffee-ring” effects, only the central region of the dried sample was used for Raman measurements, excluding edge stains (Figure S13).

Raw Raman spectra of TB at concentrations ranging from 10 μM to 100 pM are shown in full in Figure S14. At each concentration, nine independent measurements were recorded in mapping mode across 10 × 10 μm^2^ with a 5 μm step size. TB exhibited distinct characteristic peaks at 1223, 1414, 1567, and 1607 cm^–1^, shown in Figure 6A, along with a general correlation of Raman signal intensity with TB concentration. Figure 6A displays the mean Raman spectrum for each concentration with shaded regions representing standard deviations. The intensity of the 1607 and 1223 cm^–1^ peaks was plotted against concentration on a log_10_ scale (Figure 6B,C), yielding calibration curves with high linearity (*R* ^2^ = 0.97 and *R* ^2^ = 0.98, respectively). The results confirm the sensor’s capability for quantitative detection of organic dyes. Lastly, to explore measurement uniformity, Raman signal mapping was completed collecting a 5 × 5 array of data points over an area of 20 × 20 μm^2^ at 1223.09 cm^–1^, showing again uniform peak intensity across the particle film (Figure 6D), echoing that seen within PEF studies.

**Figure 6:**
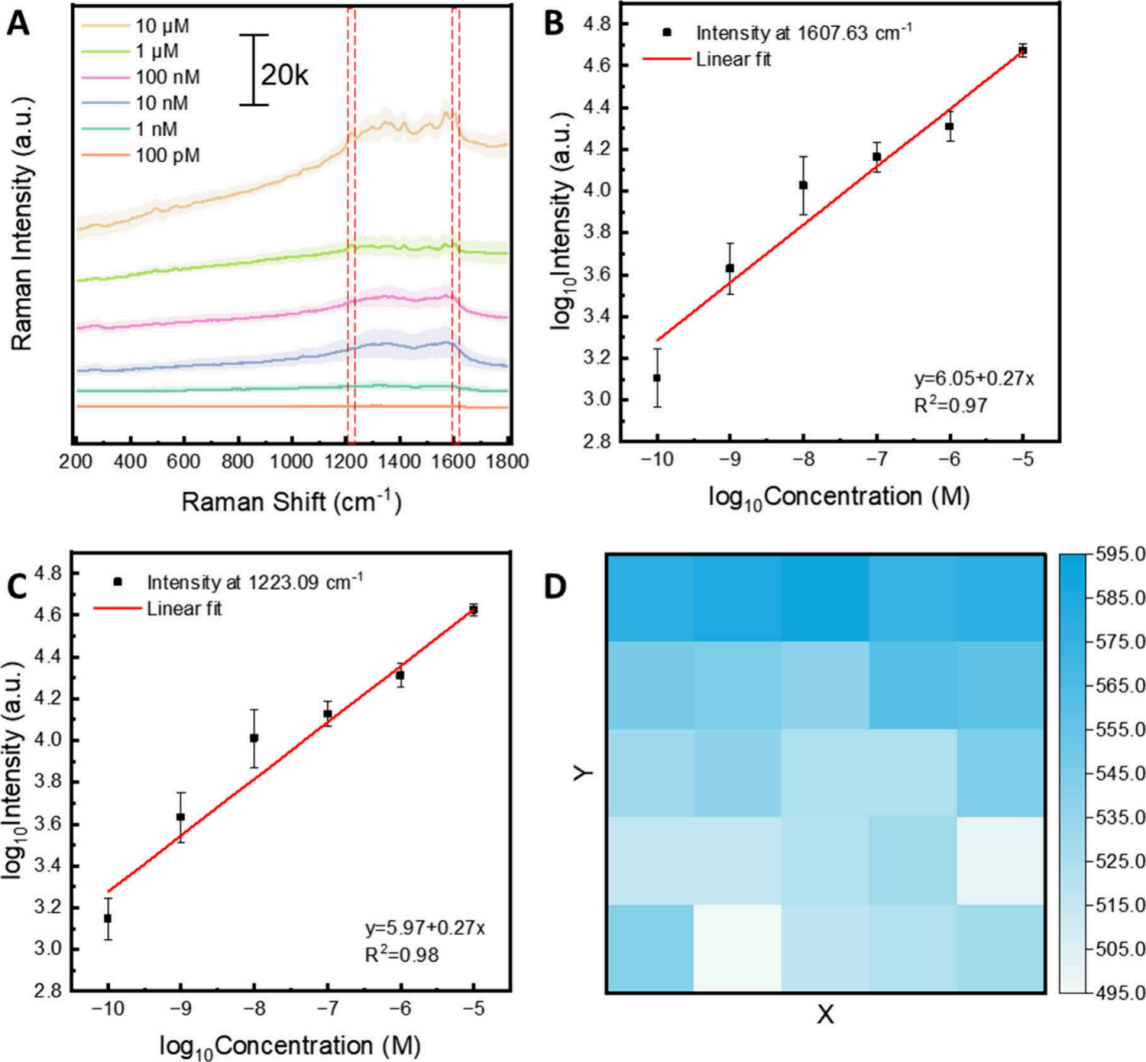
Surface-enhanced Raman scattering for quantitative detection of Trypan blue (TB) on laser processed films. TB was quantified in the concentration range of 10^–5^ to 10^–10^ M. (A) The summation of all acquired spectra at different concentrations of TB (10^–5^ to 10^–10^ M). Each spectrum represents the average value of all measurements (*n* = 9). The shaded areas represent the standard deviation of each curve. In the TB-specific windows of 1215 to 1225 cm^–1^ and 1597 to 1614 cm^–1^, two peaks were observable and used for calibration (red boxes). (B) Raman intensity versus concentration curve of TB (on a log_10_ scale) at 1607.63 cm^–1^. Error bars were calculated from *n* = 9 independent measurements. (C) Raman intensity versus concentration curve of TB (on a log_10_ scale) at 1223.09 cm^–1^. Error bars were calculated from *n* = 9 independent measurements. (D) Raman signal mapping (5 × 5 data points on an area of 20 × 20 μm^2^) at the 1223 cm^–1^ peak showing uniform intensity across the nanoparticle film.

## 3 Conclusions

Herein, we report a novel method for the fabrication and patterning of tunable plasmonic films and highlight the potential for their broadband plasmonic field enhancement, demonstrating application within PEF and surface-enhanced Raman scattering. The fabrication approach presented is a simple and scalable dual-step process that enables large-scale patterning of densely packed, highly uniform, and tunable gold nanostructures. In this article, we have investigated and characterized numerous processing conditions including the thickness of the base gold thin film layer, torch treatment time, laser processing parameters, and spacer thickness toward maximizing plasmonic enhancement. Through tuning these parameters, we have shown the ability to fabricate films with significant changes in the nanoparticle morphology and plasmonic enhancement. Moreover, through simulation studies on the fabricated nanostructures, plasmonic performance was evaluated, and the selected film progressed to experimental validation within PEF and SERS. For the selected film (T4), the spacing between the film and fluorophore was investigated, and within the optimized configuration, uniform large-area enhancement was demonstrated within both PEF and SERS applications. Together, we believe the approach presented to be a significant advancement within the field, as it overcomes limitations of current methods, including limited tunability, complex fabrication procedures, and/or difficulty in selective patterning.

In conclusion, this study outlines a promising new fabrication technique and offers areas for further investigation that could enhance applicability within biological assays. For example, all patterning within this study was completed upon glass substrates for simplicity; however, we have begun exploration on transferring fabricated particles onto “soft” substrates to enable the development of flexible plasmonic substrates. In this configuration, films could be made to be conformal and in a patch format, beneficial for PEF or SERS applications. Moreover, the ability to control patterned areas could easily be coupled with microfluidic systems to enable spatial separation of distinct analyte capture and detection areas, offering a means toward integrated assays. Thus, although promising fundamental advancements have been presented here, there is a rich area of application that remains unexplored.

## 4 Materials and Methods

### 4.1 Gold Nanoisland Thin Film Fabrication

Quartz glass slides (ESCO Optics, model R140115) were used as purchased. Gold thin films were deposited onto the glass slides using a Hummer sputter coater for 2 min at 20 mA and 60 mTorr, resulting in films that were ∼15.5 nm thick. The substrate holder was positioned at its lowest point within the chamber to increase uniformity while lowering the deposition rate of the resultant film. Once deposited, samples were flipped (Au side down) and pulse-treated (1 pulse represents 1 s of direct heat, followed by an equal dwell time to enable substrate cooling) via a MAP-GAS torch for 6–8 pulses until a visible color change in the film occurred. For initial dewetting, the sample was held between a pair of tweezers and, at a fixed distance of 100 mm away, perpendicular to the flame such that the flame clearly contacted the substrate. A notable and consistent change in the substrate between pulses could be seen with the naked eye, transforming from a dull, largely transparent thin film to an opaque reflective film. If placed on a white background, the bronze color of the initial thin film would become a deep blue color, indicating successful dewetting. Control samples included sputtered samples without torch treatment and those with rapid temperature annealing (RTA, AG Associates Heatpulse 410). For RTA-treated samples, the working temperature was kept steady at 1000 °C with a ramp rate of 100 and the time was altered from 15 to 120 s in four increments (15, 30, 60, 120 s). For experimental testing for PEF and SERS, fused quartz discs (*d* = 15 mm, thickness = 1.5 mm, Quartz Scientific Inc.) were used and processed equivalently.

### 4.2 Laser-Induced Formation of Gold Nanoparticles

Gold nanoparticles were formed upon the gold thin film samples using a Technifor Laser Marking Machine LW1 equipped with a 532 nm DPSS (diode pump solid state) laser. The working distance for all samples was fixed at 194 mm, and samples were placed Au side down upon a second glass slide. Laser parameters that were tuned included power (%), speed (mm/s), and frequency (Hz). The DPI (1200) and number of layers (1) were fixed for all tests, and raster mode was used for the laser.

### 4.3 Characterization of Gold Films

Scanning electron microscopy (SEM) images of all lased samples were completed at the Electron Microscopy facility at Dartmouth College on an FEI Helios 5CX DualBeam SEM, coated with a thin 15 nm gold layer using a Leica ACE 600 coater. The thickness of the gold films was analyzed using a stylus profilometer (KLA Tencor D-500) at a speed of 0.03 mm/s, 0.4 mm scan length, 2.5 μm range (*Z*), 0.10 mg force, average level of 16, and forward scan. Optical characterization of films was completed on a FERGIE spectrograph (Princeton Instruments) coupled to an IX-81 Olympus microscope with a 20× objective. The measurement parameters for all tests were exposure of 20 ms, center wavelength of 700 nm, and full sensor readings.

### 4.4 Simulation of Electric Field Enhancement

Lumerical FDTD was utilized to simulate the electromagnetic field enhancement of each of the fabricated structures. The structure was illuminated by a normally incident plane wave from the *z*-direction (above the substrate). Periodic boundary conditions were applied in the *x*- and *y*-directions, and perfectly matched layers (PMLs) were applied at the *z*-boundaries. The field intensity |*E*|^2^ reported here corresponds to the field enhancement factor (EF) since the input field intensity *E*_0_ = 1 for all of the simulations (EF = |*E*|^2^/|*E*_0_|^2^). Scanning electron microscopy (SEM) images of the structures were used to guide the simulations. Image processing was completed using Fiji, and the process involved thresholding images followed by making binary images for use within simulations. To smooth images prior to analysis, a median filter was also utilized on the final binary image to assimilate the smooth particle nature seen within the SEM images.

### 4.5 Plasmon-Enhanced Fluorescence

IR-125 (Exciton, Dayton, Ohio, USA) was purchased and a solution made using dimethyl sulfoxide. AlO_2_ spacer layers were deposited upon samples using atomic layer deposition (40, 80, 120, 160, and 200 cycles) to finely control the spacing between the mock agent and metal surface. For spacer optimization, a 1 μM IR-125 10 μL solution in DMSO was pipetted upon the surface, whereby after evaporation of the solvent, measurement could commence and was completed on a fluorescent plate reader (Molecular Devices SpectraMax Paradigm, 774 nm/825 nm Ex/Em). For studies using streptavidin labeled Alexa Fluor 532 dye conjugate (Invitrogen, 534 nm/553 nm Ex/Em), a linker molecule biotin-PEG-thiol (PG2-BNTH-5k, Nanocs) was used to bind streptavidin to the surface of the sensor. Briefly, the gold substrate was cleaned with DI water, followed by 100% ethyl alcohol. Then, the sample was dried and plasma-treated for 60 s using a hand-held corona surface treater (Aurora Scientific, APS-CD-20AC), after which 10 drops of a 500 μg/mL solution of biotin-PEG-thiol in DI water was pipetted on the surface and incubated for 1 h at room temperature. Next, the sample was washed with DI water, and the streptavidin solution at the desired concentration was added and left to incubate for an hour at room temperature. Finally, the sample was washed once more with DI water before each sample was placed into a 24-well plate and the fluorescence was measured on the spectrometer. All experiments were completed with *n* = 3 technical replicates, with error bars representing the standard deviation of these measurements. For well-scanning uniformity measurements, each scan number represents an individual measurement, and results are reported without averaging.

### 4.6 Surface-Enhanced Raman Spectroscopy

Raman measurements were conducted using a HORIBA XploRA Raman microspectrometer equipped with a motorized stage. The nanoparticle film was first cleaned with deionized (DI) water. A 5 μL aliquot of the aqueous sample solution was then deposited onto the film and dried at 35 °C. Once dry, the film was placed under the microspectrometer and focused using a 100× objective lens. Raman spectra were acquired point-by-point from the mapping grid using a 633 nm laser (25 mW) as the excitation source, covering a spectral range of 200–1800 cm^–1^. Each spectrum represents the average value of total *n* = 9 independent measurements, and error bars represent the standard deviation. For Raman signal mapping, measurements were collected in a 5 × 5 grid representing an area of 20 × 20 μm^2^.

### 4.7 Data Processing and Analysis

All data processing and analysis were completed using Microsoft Excel and/or MATLAB, while plotting/statistical analysis was completed using GraphPad Prism 9. All image processing and quantitative particle analysis were completed using Fiji and accompanying tools.

## Supplementary Material



## Notes

SEM images of various samples, image processing workflow, particle size analysis, optimization of laser process by altering torch treatment time, simulation workflow, 250 × 250 nm simulation results at 812 and 1046 nm, 500 × 500 nm simulation results at 562 nm, FDTD simulations of 5 nm Au nanoislands on glass, ALD processing ellipsometry measurements for film thickness, “coffee-ring” effect, Raman spectra of TB at various concentrations collected by the nanoparticle film, and laser parameters for various trials explored in this study (PDF)
