# Supplementary material for: Rapid, Tunable, and Scalable Patterning of Plasmonic Films for Biosensing Applications
Source: ACS Appl Mater Interfaces. 2025 Sep 1;17(36):50227–36. doi: 10.1021/acsami.5c09086 (PMC12442006; doi:10.1021/acsami.5c09086)
Supplement: Supplementary file 1 [file am5c09086_si_001.pdf]

Supporting Information:

Rapid, tunable, and scalable patterning of plasmonic films for biosensing applications

Authors: John H. Molinski[1], Junhu Zhou[1], Tim Palinski[2], John X.J. Zhang[1,3*]

1: Thayer School of Engineering, Dartmouth College, 15 Thayer Drive, Hanover, NH, 03755, USA
2: Honeywell International Inc, 303 S Technology Ct, Broomfield, CO, 80021, USA
3: Norris Cotton Cancer Center, Dartmouth Hitchcock Medical Center, 1 Medical Center Dr, Lebanon, NH 03766, USA

*Corresponding author.

Mailing address: Thayer School of Engineering at Dartmouth, 15 Thayer Drive, Hanover, NH, 03755, USA.

Phone: 1-603-646-8787**. E-mail:** john.zhang@dartmouth.edu.s

Figures S1-S14 & Table S1

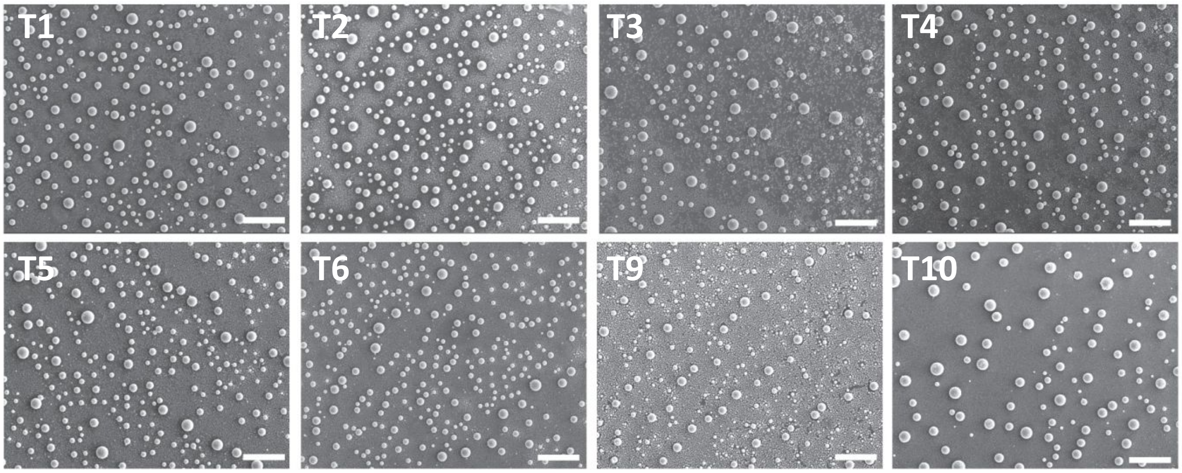

Figure S1: SEM images showing laser processed samples without torch treatment. Scale bars: 1 μm.

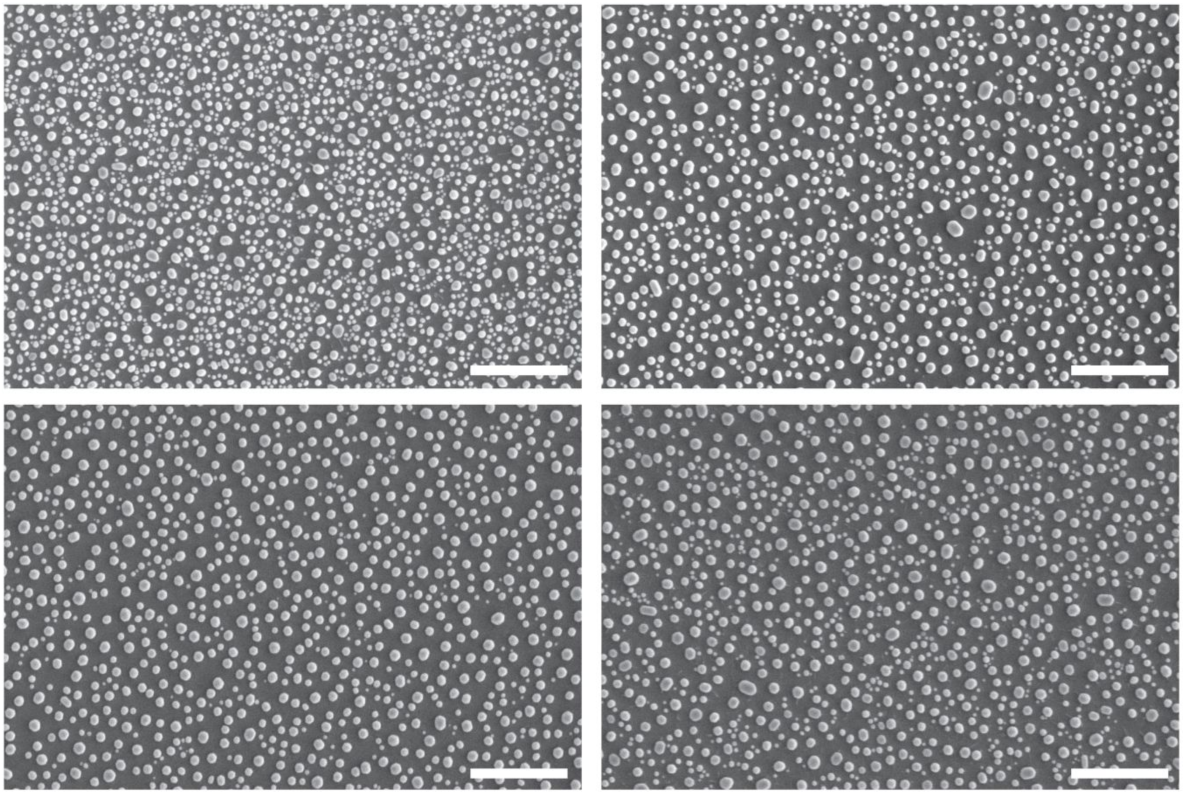

Figure S2: Rapid temperature annealing of films with same gold thickness as those utilized for basis of this study. Images show SEM images with increasing time of RTA treatment (15s – top left, 30s - top right, 60s - bottom left, and 120s – bottom right), all ramping to 1000˚C was completed with a ramp rate of 100. Scale bars: 1 μm.

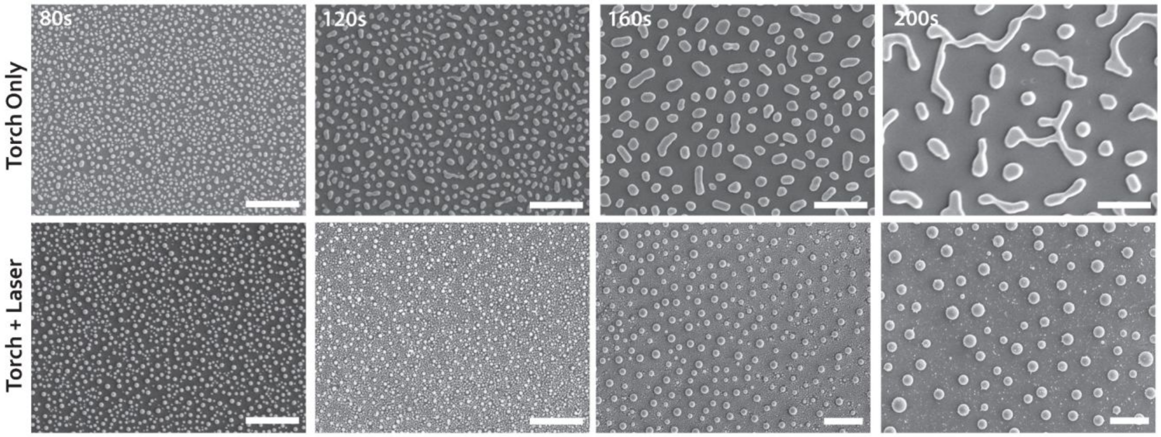

Figure S3: Optimization of laser process via altering initial gold thin film thickness. Top row shows SEM images for the various torched films of various deposition times (L to R: 80s, 120s, 160s, 200s) showing increase in particle size and decrease in particle density with increasing film thickness. Bottom row shows torched films following laser treatment using T4 processing conditions, showing corresponding increase in particle size as a result of thicker base films. Scale bars: 1 μm.

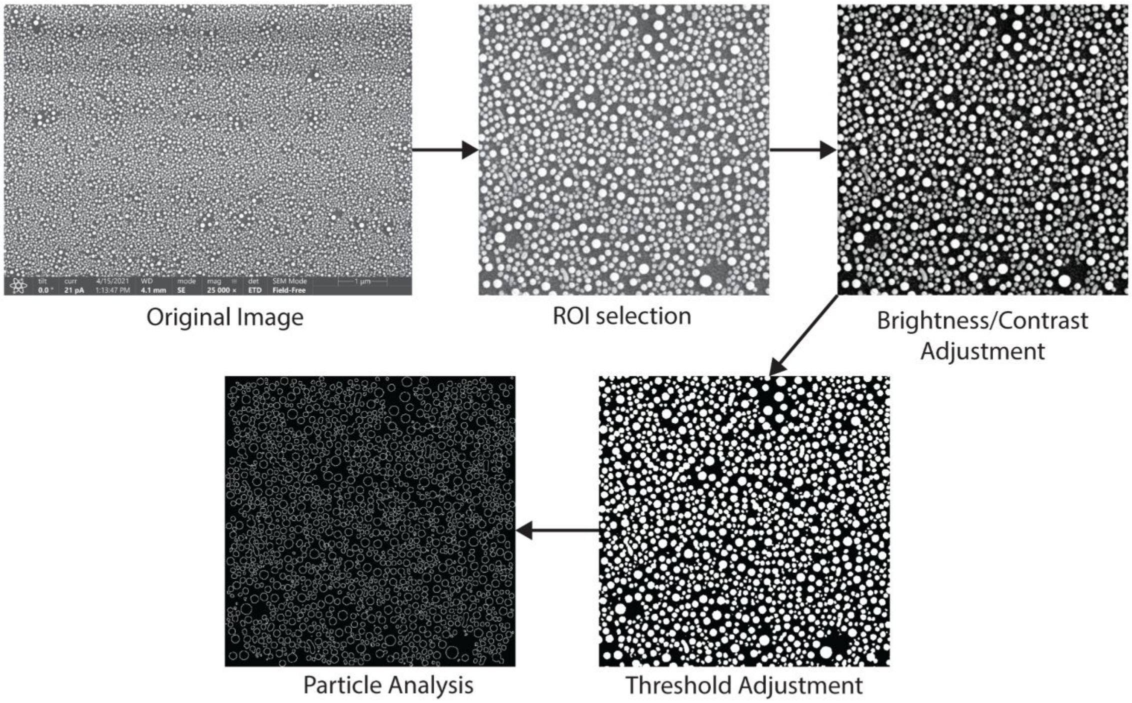

Figure S4: Image processing workflow for particle size and areal density calculation using various SEM images collected from various trials. For simulation studies, workflow was largely the same aside from addition median filter imposed on images following threshold adjustment.

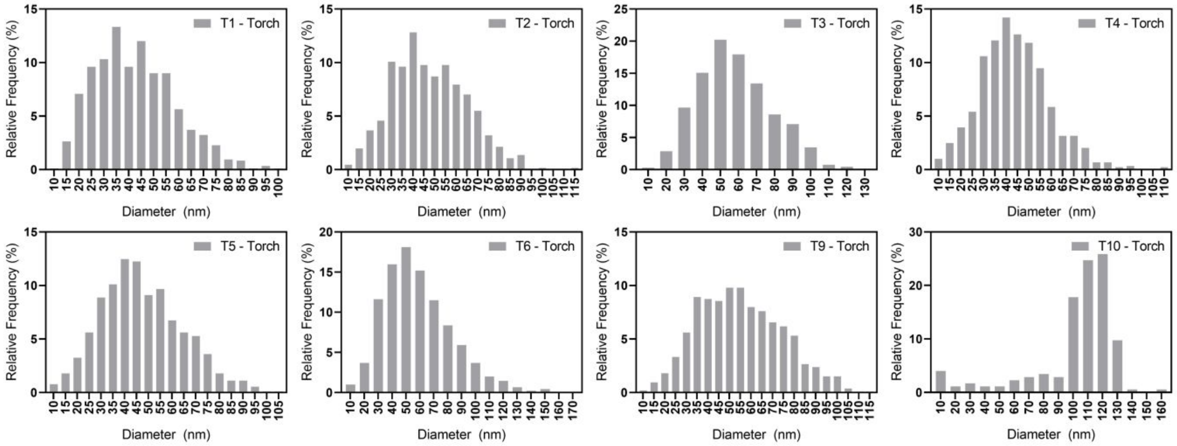

Figure S5: Histograms for particle size analysis for each of the selected laser parameters.

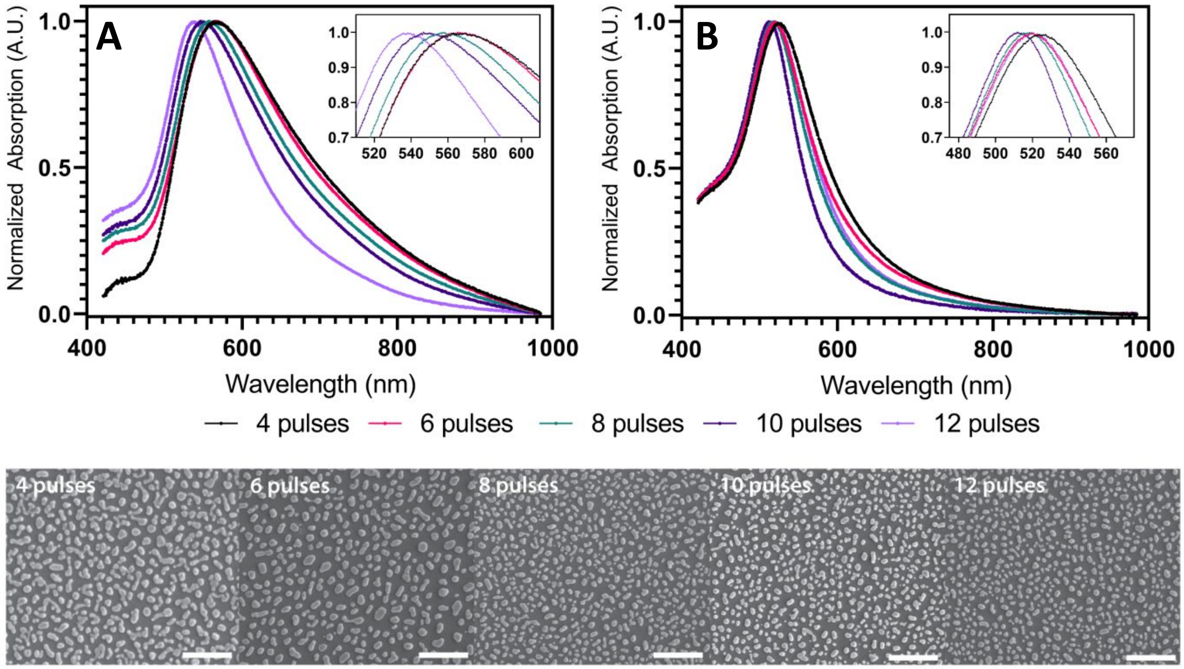

Figure S6: Optimization of laser process via altering time torched. A) Shows spectrometer measurements following various levels of torch treatment from 4-12 pulses. B) Shows corresponding spectrometer measurements for same torched samples followed by an identical laser treatment (T4) highlighting little influence on torch time on final particle formation. Below shows row of SEM images following each of the torch treatments, highlighting changes in morphology that correlate with slight changes in optical spectra. Scale bars: 1 μm.

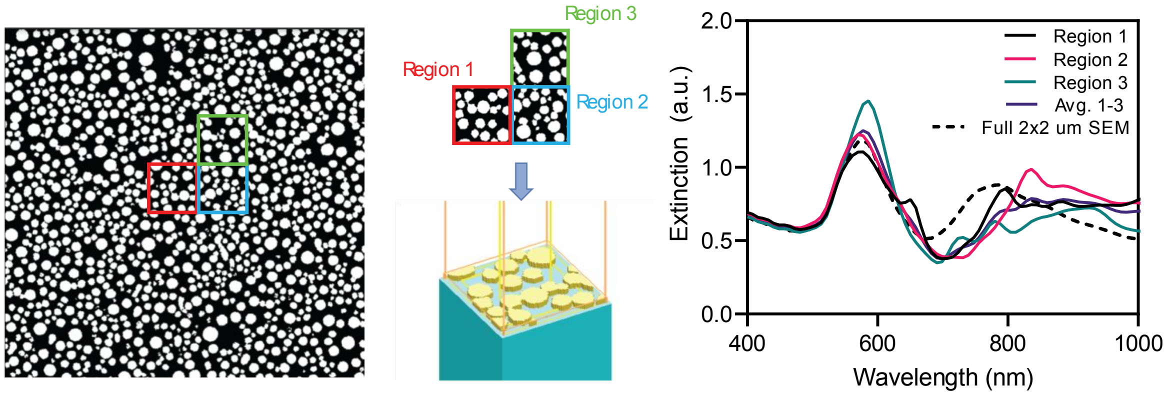

Figure S7: Simulation workflow and rational for simulation size selected.

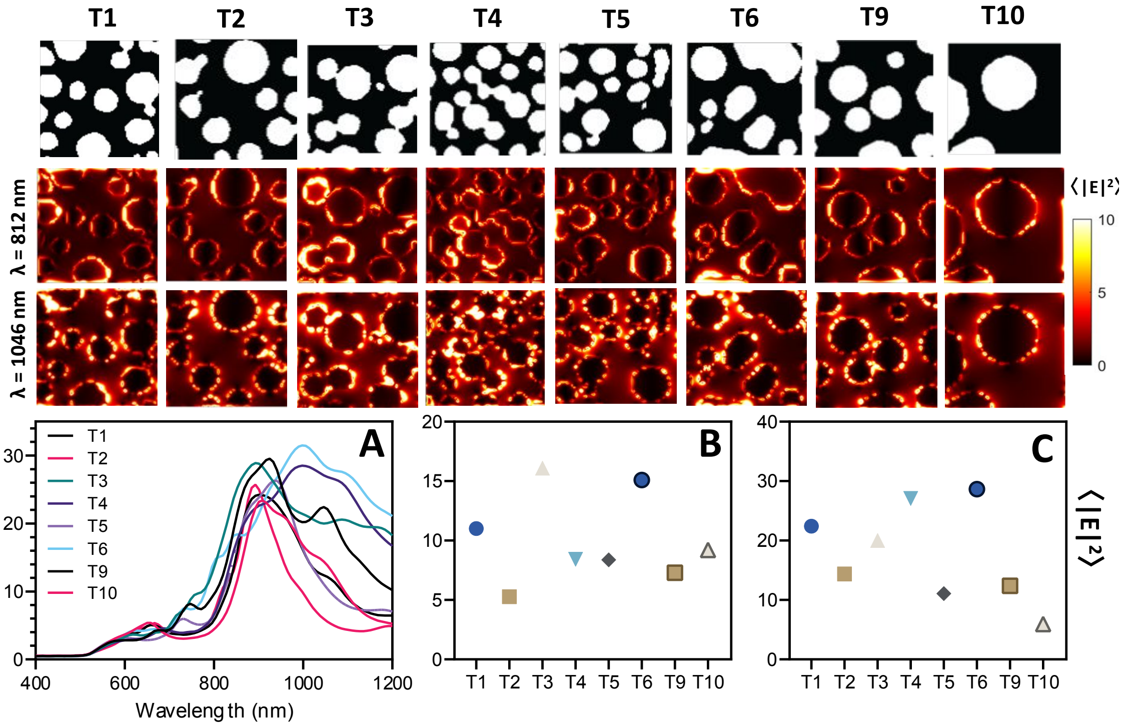

Figure S8: 250 nm x 250 nm simulation results for each laser parameter tested, at wavelengths of 812, and 1046 nm, respectively. A) Shows electric field enhancement spectrum as a function of wavelength, where B and C shows magnitude as two distinct wavelengths, namely 812 nm and 1046 nm.

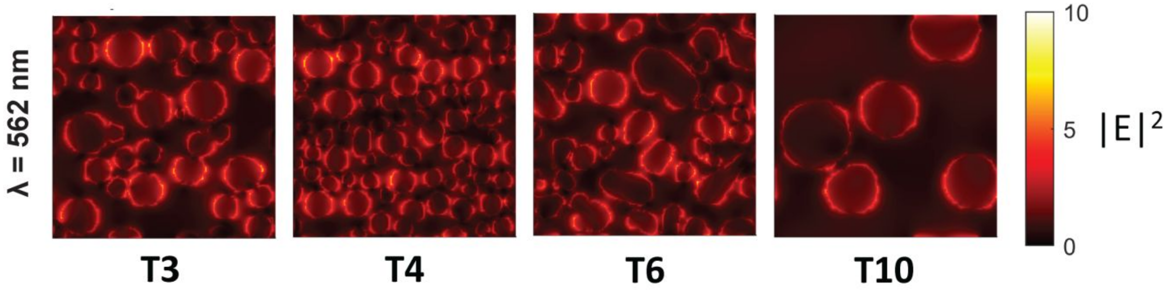

Figure S9: 500 x 500 nm simulation of T3, T4, T6 and T10, at λ=562 nm, showing significantly lower levels of enhancement compared to near-IR regime.

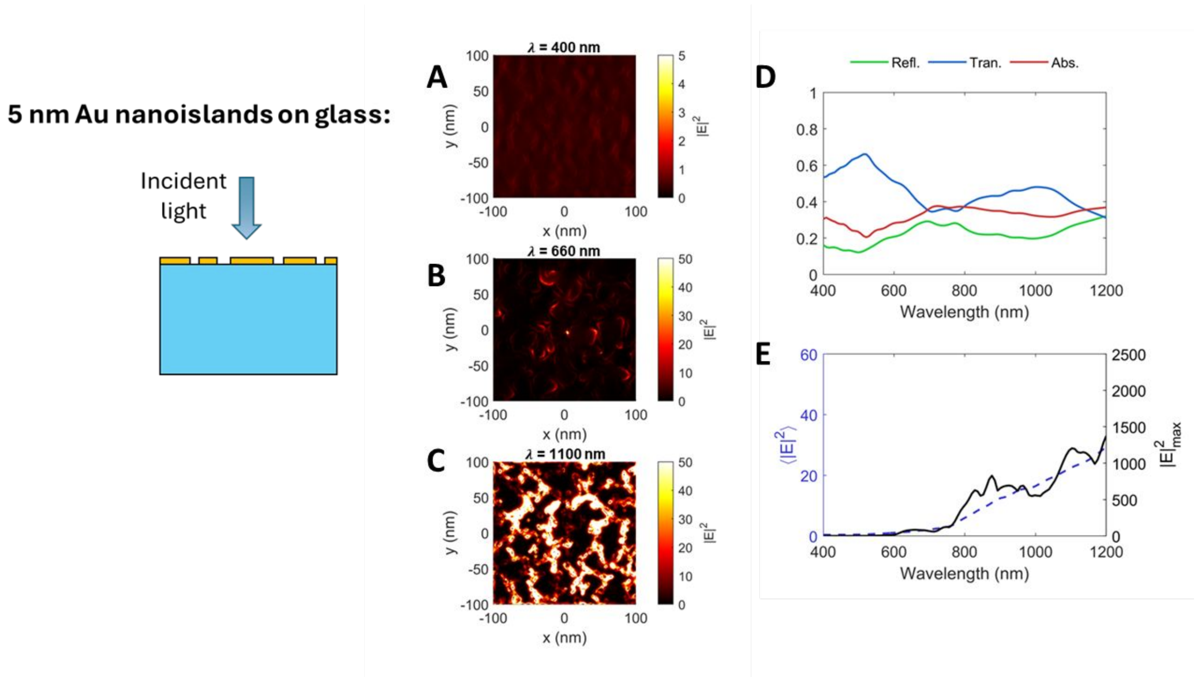

Figure S10: FDTD simulations of 5 nm Au nanoislands on glass at A) 400, B) 660, and C) 1100 nm. D) Simulated reflectance, transmittance, and absorption spectrum. E) Electric field enhancement as a function of wavelength.

36

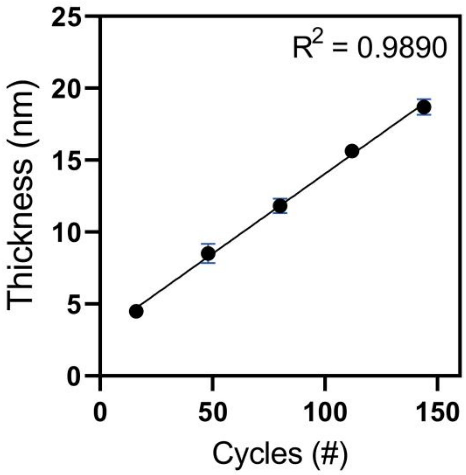

Figure S11: ALD processing ellipsometry measurements for film thickness.

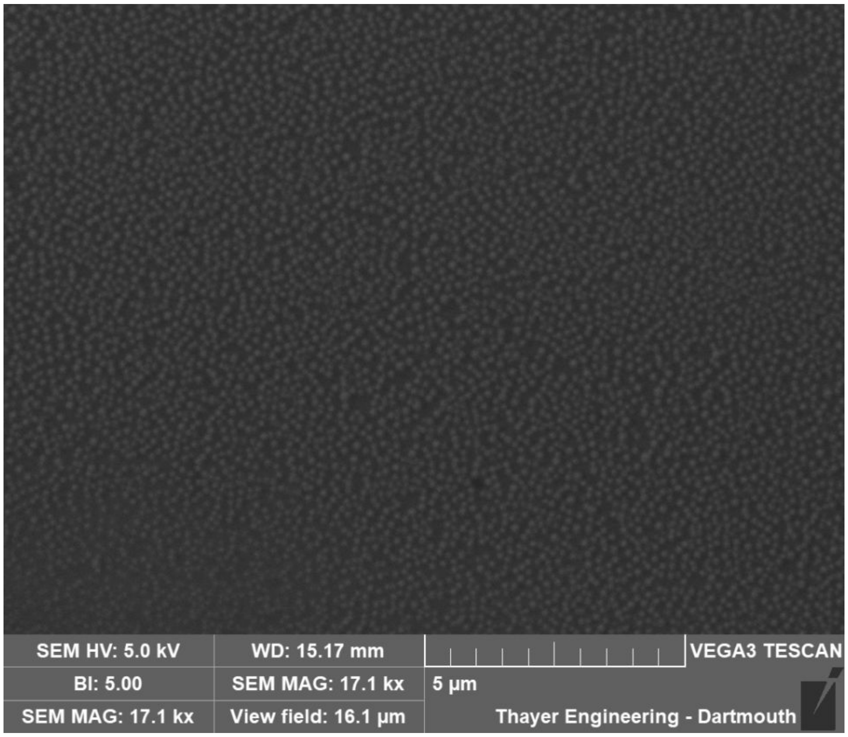

Figure S12: Scanning electron microscopy image of 12 nm aluminum oxide layer deposited upon T4 processed substrate, showing no distinct changes in morphology after adding spacer layer.

42

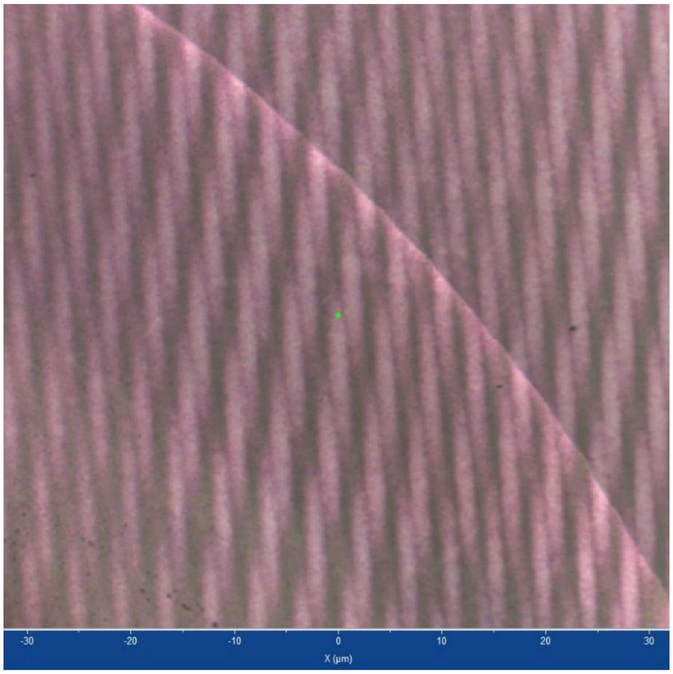

Figure S13: **"**Coffee-ring" effect of liquid sample on the nanoparticle film. The Raman measurements were taken at the center to avoid nonuniform analyte distribution.

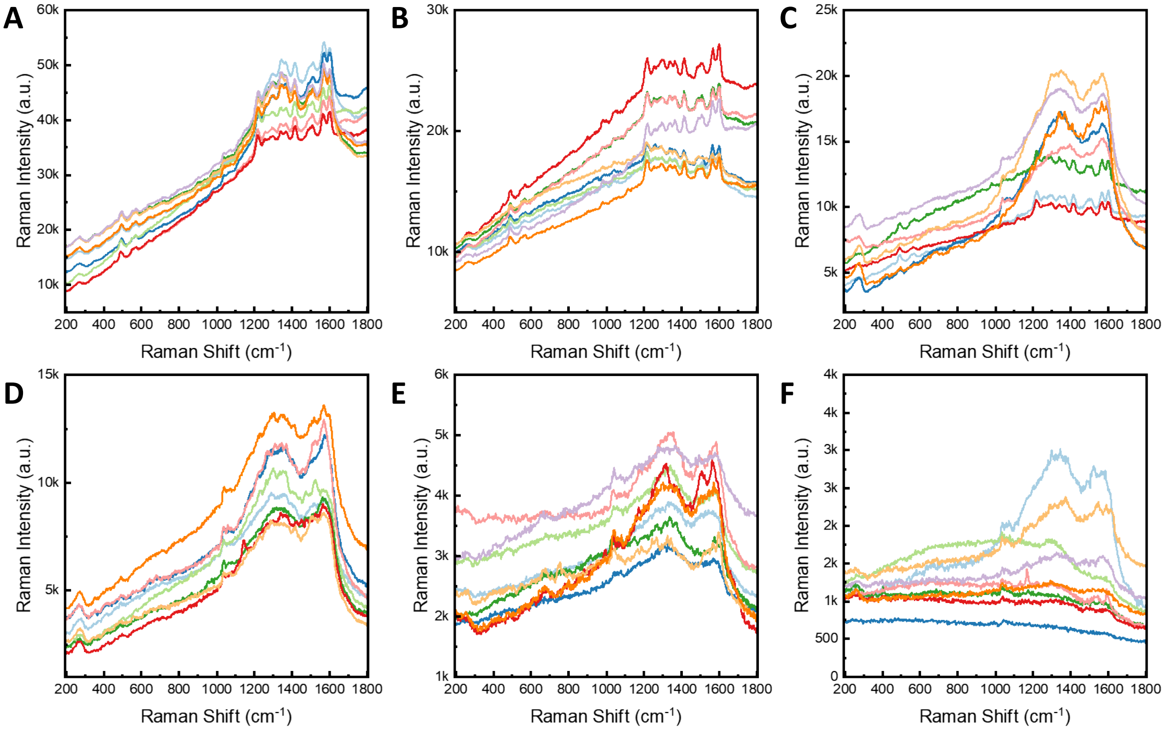

Figure S14: Raman spectra of TB collected by the nanoparticle film in the concentration range of 10^{-5} to 10^{-10} M. Each group had 9 independent measurements. A) Raman spectra of TB at 10^{-5} M. B) Raman spectra of TB at 10^{-6} M. C) Raman spectra of TB at 10^{-7} M. D) Raman spectra of TB at 10^{-8} M. E) Raman spectra of TB at 10^{-9} M. F) Raman spectra of TB at 10^{-10} M.

Trial #	Power (%)	Speed (mm/s)	Frequency (Hz)	# of passes	DPI	Time for in^2
T1	***2***	***200***	***100k***	***1***	***1200***	***2m 34s***
T2	***4***	***200***	***100k***	***1***	***1200***	***2m 34s***
T3	***8***	***200***	***100k***	***1***	***1200***	***2m 34s***
T4	***2***	***100***	***100k***	***1***	***1200***	***5m 7s***
T5	***2***	***300***	***100k***	***1***	***1200***	***1m 43s***
T6	***2***	***500***	***100k***	***1***	***1200***	***1m 2s***
T7	2	200	30k	1	1200	2m 34s
T8	2	200	60k	1	1200	2m 34s
T9	***2***	***200***	***90k***	***1***	***1200***	***2m 34s***
T10	***1***	***50***	***100k***	***1***	***1200***	***10m 12s***
T11	10	500	100k	1	1200	1m 2s
T12	1	50	50k	1	1200	10m 12s
T13	10	500	50k	1	1200	1m 2s

Table S1: Laser parameters for various trails explored within this study. Trials T7, T8, T11-T13 omitted from study due to lack of particle formation
